# Comparison of dipole-based and potential-based ECGI methods for premature ventricular contraction beat localization with clinical data

**DOI:** 10.3389/fphys.2023.1197778

**Published:** 2023-06-09

**Authors:** Yesim Serinagaoglu Dogrusoz, Nika Rasoolzadeh, Beata Ondrusova, Peter Hlivak, Jan Zelinka, Milan Tysler, Jana Svehlikova

**Affiliations:** ^1^ Department of Electrical-Electronics Engineering, Middle East Technical University, Ankara, Türkiye; ^2^ Department of Scientific Computing, Middle East Technical University, Institute of Applied Mathematics, Ankara, Türkiye; ^3^ Institute of Measurement Science, Slovak Academy of Sciences, Bratislava, Slovakia; ^4^ Faculty of Electrical Engineering and Information Technology, Slovak University of Technology, Bratislava, Slovakia; ^5^ National Institute for Cardiovascular Diseases, Bratislava, Slovakia

**Keywords:** electrocardiographic imaging (ECGI), inverse problem of electrocardiography (ECG), premature ventricular contraction (PVC), torso inhomogeneities, regularization

## Abstract

**Introduction:** Localization of premature ventricular contraction (PVC) origin to guide the radiofrequency ablation (RFA) procedure is one of the prominent clinical goals of non-invasive electrocardiographic imaging. However, the results reported in the literature vary significantly depending on the source model and the level of complexity in the forward model. This study aims to compare the paced and spontaneous PVC localization performances of dipole-based and potential-based source models and corresponding inverse methods using the same clinical data and to evaluate the effects of torso inhomogeneities on these performances.

**Methods:** The publicly available EP solution data from the EDGAR data repository (BSPs from a maximum of 240 electrodes) with known pacing locations and the Bratislava data (BSPs in 128 leads) with spontaneous PVCs from patients who underwent successful RFA procedures were used. Homogeneous and inhomogeneous torso models and corresponding forward problem solutions were used to relate sources on the closed epicardial and epicardial–endocardial surfaces. The localization error (LE) between the true and estimated pacing site/PVC origin was evaluated.

**Results:** For paced data, the median LE values were 25.2 and 13.9 mm for the dipole-based and potential-based models, respectively. These median LE values were higher for the spontaneous PVC data: 30.2–33.0 mm for the dipole-based model and 28.9–39.2 mm for the potential-based model. The assumption of inhomogeneities in the torso model did not change the dipole-based solutions much, but using an inhomogeneous model improved the potential-based solutions on the epicardial–endocardial ventricular surface.

**Conclusion:** For the specific task of localization of pacing site/PVC origin, the dipole-based source model is more stable and robust than the potential-based source model. The torso inhomogeneities affect the performances of PVC origin localization in each source model differently. Hence, care must be taken in generating patient-specific geometric and forward models depending on the source model representation used in electrocardiographic imaging (ECGI).

## 1 Introduction

Cardiovascular diseases are among the leading causes of death in the world (EUROSTAT, 2019). Therefore, noninvasive diagnosis of these diseases and development of effective treatment strategies have been a top priority for clinicians and researchers. Abnormal electrical activity of the ventricles can result in various types of arrhythmias, from single premature ventricular contractions (PVCs), caused by early activation of the ventricles, usually from a small single location in the ventricles, to sustained ventricular tachycardia. These arrhythmias can later lead to heart failure and decreased pumping performance/output of the left ventricle.

PVCs usually start in a small single location of the ventricles, unusual for normal activation. This ectopic activity of the heart can sometimes be treated pharmacologically. However, in many cases, such treatment is not effective. Radiofrequency catheter ablation (RFA) is an effective but highly invasive and time-demanding procedure to treat these arrhythmias (Hlivák et al., 2011). This method is based on leading the catheter to the right or the left ventricle, mapping the endocardial electrical activity, and applying a high-frequency electric current to the estimated PVC origin to suppress these premature beats. The catheter approach to the right and left ventricles differs. Therefore, the intervention procedure can be considerably prolonged if an initial survey of the PVC origin starts in the “wrong” ventricle. Electrocardiographic imaging (ECGI) provides a preliminary location of the origin of the ectopic activity. Thus, it is a promising tool for guiding the RFA procedure and shortening its duration (Sapp et al., 2012; Erem et al., 2014a; Potyagaylo et al., 2019b; Duchateau et al., 2019; Zhou et al., 2019).

In ECGI, the inverse problem of electrocardiography (ECG) is solved to reconstruct the electrical activity of the heart based on noninvasive measurements of the body surface potentials (BSP) and a patient-specific model of the heart and torso geometry. However, due to the potential attenuation and smoothing effect of the thorax, this inverse problem is ill-posed, meaning that the solution may not be unique, and it is very sensitive to noise in the measurements (Gulrajani, 1998; Cluitmans et al., 2018). To overcome this ill-posedness, regularization should be applied to stabilize the solution by imposing additional constraints. The success of ECGI strongly depends on the prior assumptions/constraints and the applied regularization method. Different research groups have introduced various methods based on the target clinical application in mind and the choice of the equivalent cardiac source model (see (Gulrajani, 1998; Cluitmans, 2015; Cluitmans et al., 2018; Bergquist J. et al., 2021) for detailed reviews).

ECGI has been formulated in terms of various equivalent cardiac source models, including single/multiple equivalent dipoles or equivalent double-layer (EDL) representation (Zhou et al., 2019; Svehlikova et al., 2022), activation isochrones (Erem et al., 2014b; Potyagaylo et al., 2019b), transmural or heart surface transmembrane potentials (Wang et al., 2011; Rahimi et al., 2016; Schuler et al., 2022), and extracellular potentials on the closed epicardial (“Epi”) or combined epicardial/endocardial (‘EpiEndo’) heart surface (Bear et al., 2019; Erenler and Serinagaoglu Dogrusoz, 2019; Onak et al., 2022; Schuler et al., 2022). In this study, we compare two well-established cardiac source models for PVC localization: a single equivalent dipole-based source model and the model in terms of extracellular potentials defined on the heart surface (Epi and EpiEndo), which is a monopole-based source model coupled with BEM. The former finds a single equivalent dipole that best fits the BSP measurements in the early intervals of the premature QRS complex. The PVC location is thus a direct result of this method. However, if one is interested in the electrical activity of the heart over the whole cardiac cycle, potential-based solutions become a better choice. On the other hand, PVC localization from the potential-based approach requires finding the activation times (AT) and determining the earliest activated location. These post-processing steps could introduce artifacts in the reconstructed activation isochrones, as demonstrated in Schuler et al. (2022), which could cause errors in the PVC location estimates.

ECGI has been extensively evaluated individually in torso-tank experiments using excised hearts (Bear et al., 2018; Bear et al., 2019; Bergquist J. A. et al., 2021) and *in situ* animal experiments (Cluitmans et al., 2017; Burton et al., 2018). In recent years, there has been growing interest in the clinical application of ECGI (Cluitmans et al., 2018; Bergquist J. et al., 2021), most commonly for pacing site/PVC origin localization. In some of these studies, ECGI is applied to patients undergoing electrophysiologic mapping and ablation treatments, where the ground truth is defined in terms of invasive mapping procedure recordings (CARTO, Biosense Webster Inc., Diamond Bar, CA; EnSite NavX–St. Jude Medical, Saint Paul, MN; RHYTHMIA–Boston Scientific Inc., Natick, MA; NEEES, EP Solutions SA, Yverdon-les-Bains, Switzerland) (Sapp et al., 2012; Erem et al., 2014a; Tsyganov et al., 2017; Duchateau et al., 2019; Zhou et al., 2019). Other studies validated ECGI methods for patients with implantable CRT devices or patients whose hearts were paced via the catheter tip during the ablation procedure; thus, the location of the pacing electrode is the ground truth (Potyagaylo et al., 2019b). These studies used Epi or EpiEndo potentials and equivalent double layer (EDL) as the equivalent cardiac source model. To our knowledge, there has been no comprehensive comparison of the dipole-based and potential-based solutions in their ability to estimate the origins of the paced and naturally occurring PVCs with clinical data.

Another limitation of the ECGI studies is the level of complexity assumed in the forward model calculation. There have been several studies on the effects of torso inhomogeneities on forward and inverse solutions. Bear *et al.* concluded that including the inhomogeneities improved the forward-computed BSPs (Bear et al., 2015). Ramanathan *et al.* argued that there was no significant difference in inverse solutions when the homogeneous torso model was used (Ramanathan and Rudy, 2001b). On the other hand, van Oosterom claimed that the solutions improved when the lungs were included in the torso model (van Oosterom, 2014). Zemzemi *et al.* showed that the effects of torso inhomogeneities on the inverse solutions depended on the measurement noise level, which was more influential with small measurement errors and less significant when the noise gets higher (Zemzemi et al., 2016). Punshchykova *et al.* reported the most stable results with the homogeneous model, but fidelity to the ground truth was best when the most complex inhomogeneous model was used (Punshchykova et al., 2016). ECGI methods that are based on comparing the measured BSPs with computed BSPs simulated from candidate cardiac source representations seek more realistic torso models. Potyagaylo et al. investigated the influence of cardiac source and torso modeling errors using simulated data (Potyagaylo et al., 2016). Their torso models included blood cavities, lungs, and liver and showed that their proposed inverse method was robust to the model errors. The conflicting results observed in different studies demonstrate the need for further investigation of the effects of torso inhomogeneities on PVC localization performance.

This study aims to

• Apply ECGI methods for localizing the origins of (1) paced beats in patients with previously implanted CRT devices and (2) spontaneous PVCs in patients who underwent RFA procedures.
• Evaluate and compare the pacing site/PVC origin localization performances of single dipole-based and heart surface potential-based (Epi/EpiEndo) models/methods on the same clinical data set.
• Study the effects of torso inhomogeneities (lungs and/or blood) on the performances of pacing site/PVC origin localization in each source model.

A publicly available data set with known pacing sites based on implanted CRT devices and clinical data of patients with PVC diagnosis who have undergone invasive endocardial mapping and RFA procedures have been used. Preliminary results were presented in Rasoolzadeh et al. (2022), in which PVC localization performances of different torso models have been evaluated qualitatively on five patients. Here, the data set was enhanced by including 10 patients with spontaneous PVC and the aforementioned paced data set. Quantitative as well as qualitative evaluations were performed based on ground truth pacing site/PVC origin locations.

## 2 Methods

### 2.1 Data sets

#### 2.1.1 EDGAR EP-Solutions data

The first data set used in this study is obtained from the publicly available EDGAR database of the Consortium for Electrocardiographic Imaging (https://www.ecg-imaging.org/) (Aras et al., 2015). Detailed information on this data set and how the study was conducted can be found in Potyagaylo et al. (2019b); the study was reviewed and approved by the Ethical Committee of Almazov National Medical Research Center in Saint Petersburg, Russia.

Data from five patients were used in the study. For three of them, the BSPs from both LV and RV pacing locations were provided. For the other two patients, only the RV-paced data were used. Thus, altogether, eight well-defined data sets were analyzed. BSPs were measured using the multichannel Amycard 01C EP system ECG amplifier (EP Solutions SA, Switzerland) via a maximum of 240 electrodes. During data acquisition, a CRT device in each patient provided isolated RV/LV pacing from implanted leads at a rate not more than 90 bpm for a duration of 10 s.

Immediately after the BSP measurements, cardiac CT scans of these patients were obtained with the measuring electrodes still attached to their bodies. These CT images were then segmented to construct patient-specific geometric models using the Amycard 01C EP system software. For this set, only the homogeneous torso model was available; inhomogeneities were not provided as part of the torso geometries of patients. The heart geometry was modeled as an EpiEndo surface. This process resulted in 1395 ± 7 fine torso nodes and 1916 ± 302 EpiEndo surface nodes. The ground truth about the origin of the pacing stimulus was defined from the CT scan as the tip of the stimulating electrode.

#### 2.1.2 Bratislava data

Data were acquired from 10 patients at the National Institute for Cardiovascular Diseases (NICD) under the supervision of the physician (Svehlikova et al., 2022). The measurements were approved by the Ethical Committee of the NICD. Written informed consent was obtained from each patient after a detailed description and explanation of the study before the procedures. This study was performed following the Good Clinical Practice guidelines and Helsinki Declaration for biomedical research. Patients selected for this study had spontaneous PVCs indicated for RFA, and their information is summarized in Table 1.

**TABLE 1:** Patient information for the Bratislava data set. Note that P5 was ablated on both sides of the septum.

Patient #	Age	Sex	Meas–RFA interval (days)	Ablation side
P1	17	M	180	RVOT
P2	63	M	282	RVOT
P3	72	F	5	RVOT
P4	46	M	2	RVOT
P5	59	M	330	Septum (LV/RV)
P6	37	M	42	RV
P7	33	M	2	LV
P8	38	M	380	RVOT
P9	64	F	0	RVOT
P10	61	M	150	LV

**FIGURE 1:**
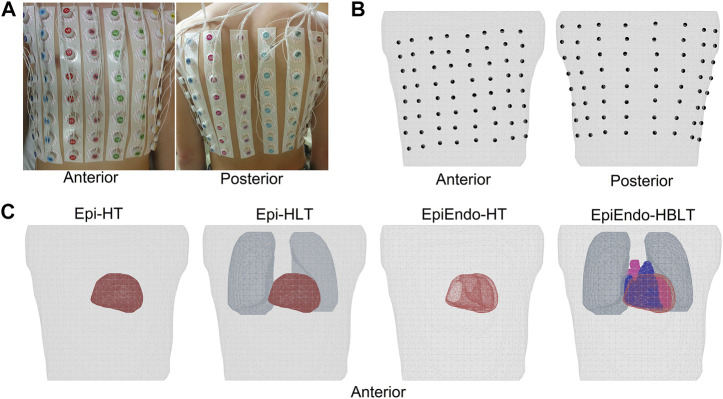
**(A, B)** Top row showing the anterior and posterior views of the body with the electrodes attached and the mesh generated from the body with the electrode positions marked on the torso surface, respectively. Four different heart–torso geometries used in this study are given in **(C)** of the bottom row. Homogeneous models only include the heart and torso surfaces (Epi–HT and EpiEndo–HT). Lungs are included in the Epi inhomogeneous model (Epi–HLT), and both blood and the lungs are included in the inhomogeneous EpiEndo model (EpiEndo–HBLT).

BSPs were measured at a 1,000 Hz sampling rate for a duration of 5–20 min using the ProCardio-8 measuring system (Kadanec et al., 2017). This acquisition system uses 133 ECG electrodes. A total of 128 disposable electrodes were placed around the chest, organized in 16 strips of eight electrodes, as shown in Figure 1A. The standard limb-lead electrodes were placed on both arms and on the left leg. The active grounding electrode was placed on the right leg (driven right leg—DRL). The reference electrode (common mode sense—CMS) was ideally placed in the center of the measuring electrodes.

Immediately after the BSP measurements, CT scans of the torso regions of these patients were obtained with the measuring electrodes still attached to their bodies for generating patient-specific torso geometric models. These CT images were segmented into the epicardium, torso surface, and the main inhomogeneities such as the heart cavities and lung lobes, using the commercial segmentation software TOMOCON (https://tatramed.sk/tomocon-workstation/). This software also generates triangulated surface meshes of these segmented regions, resulting in 3434 ± 455 nodes for the fine torso, 1794 ± 340 nodes for the epicardium, 3764 ± 741 nodes for EpiEndo, 1417 ± 326 nodes for the lungs, and 3293 ± 338 nodes for the blood cavities. Finally, the positions of the electrodes were extracted from the CT images to map the measurement locations to the patient-specific geometry. The electrode positions marked on the torso surface mesh and various heart–torso geometries used in this study are given in Figures 1B, C, respectively.

These patients then underwent endocardial mapping and the RFA procedure, and the origin of the undesired ventricular activity was found invasively. Therefore, for all patients, information about the successful position of the ablation intervention was available for validation of the results. The time between the BSP measurement and the invasive intervention varied from 0 to 380 days, as listed in Table 1.

### 2.2 Pre-processing of the BSPs

Details of the pre-processing of all ECG leads were discussed previously in Svehlikova et al. (2022). A summary of these steps is given as follows.

The measured ECG signals were high-pass filtered using a finite impulse response filter with a cut-off frequency of 0.5 Hz designed by the windowing method (Blackman–Harris) to remove the baseline drift wandering. In a chosen reference lead (usually ECG lead II), R-peaks were found for all cardiac cycles, and the cycles were defined by the proper time intervals around the R-peak. Then, the cycles were clustered according to their morphology, and the signals in the clusters with PVC morphology were averaged. Finally, the QRS onset of the PVC beat was estimated manually, and the baselines in all leads were corrected by a constant value such that at this onset, the BSP value became 0. This signal-averaged PVC beat was then used for ECGI.

### 2.3 Inverse problem of ECG

In this study, we evaluated two different equivalent source models: dipole-based and potential-based. Solutions were reconstructed over closed Epi or EpiEndo heart surfaces. A brief description of these source models and the methods used for solving the forward and inverse problems of ECG is given below.

#### 2.3.1 Dipole-based inverse solution

PVC starts at a single position in the ventricles. Therefore, at the beginning of the activation during the first 20–30 m, we can presume that the activated area is small enough to be represented by a single dipole (Svehlikova et al., 2018). Considering the torso as a volume conductor surrounded by a non-conductive medium, for a dipole in a specific position in the heart, BSPs are computed by the linear equation:

$$y\left(k\right)={Td}_{i}\left(k\right)+v\left(k\right),\;k=1,2,\ldots ,K,$$ (1)

where **T** is a transfer matrix computed by the boundary element method (BEM) for a torso model, describing the relation between the dipole **d**_*i*_(*k*) in the given position *i*, and the corresponding potentials on the torso surface **y**(*k*), at time instant *k*, and **v**(*k*) represents noise in the measurements. Then, for the unknown dipole moments, Eq. 1 leads to

$${\hat{d}}_{i}\left(k\right)=T^{†}y\left(k\right)\;.$$ (2)

Here, **T** ^†^ is the pseudo-inverse of matrix **T**, and Eq. 2 has a unique solution fulfilling the criterion of the minimal least-squares method. Regarding the presumption of a small activated area, Eq. 2 (inverse solution) is computed only during the initial time interval up to 30 m of the PVC signal.

Possible positions of the PVC origin *i* (*i* = 1, 2, … , *N*, where *N* is the number of nodes/possible dipole locations on the heart) are defined on the whole surface or in the whole volume of the ventricular model. In this study, closed Epi and EpiEndo surface models were used, and the possible positions of the inverse dipole were defined on the vertices of these triangulated surfaces. Then, the inverse solution is computed for each predefined position *i* and each time instant *k* starting from the beginning time interval. The quality of each result is evaluated by the relative residual error (RRE) between the measured potentials **y**(*k*) and those computed using the inversely estimated dipole $\left(y_{\mathrm{comp}}^{i}\left(k\right)={T\hat{d}}_{i}\left(k\right)\right)$ :

$${RRE}^{i}\left(k\right)=\frac{\left\|y\left(k\right)-y_{\mathrm{comp}}^{i}\left(k\right)\right\|}{\left\|y\left(k\right)\right\|}.$$ (3)

Here, ‖.‖ is the Euclidean norm. Then, the position *i* of the dipole with the minimal **RRE** ^*i*^(*k*) value out of all time instants and all positions on the ventricular surface is assigned as the location of the PVC origin.

#### 2.3.2 Potential-based inverse solution

In this formulation, the electrical activity of the heart is represented in terms of extracellular potentials on the heart surface (Epi or EpiEndo). These surface potentials are linearly related to the corresponding BSPs:

$$y\left(k\right)=Ax\left(k\right)+v\left(k\right),\;k=1,2,\ldots ,K,$$ (4)

where $x\left(k\right)\in R^{N\times 1}$ are the heart surface potentials and $y\left(k\right)\in R^{M\times 1}$ are the corresponding BSPs at time instant *k*. $A\in R^{M\times N}$ is the forward transfer matrix, and **v**(*k*) represents noise in the measurements. Depending on the source model, **x**(*k*) corresponds to either the epicardial surface or the EpiEndo surface potentials. The matrix **A** is computed by solving the forward problem of ECG using the BEM (Barr et al., 1977; Stanley, 1986).

The potential-based inverse problem is highly ill-posed, and even small amounts of noise in the measurements yield large errors in the solution. Here, we used the Tikhonov regularization method (Tikhonov and Arsenin, 1977) to deal with this ill-posedness. This method minimizes a cost function that seeks a trade-off between a fit to the measurements and a good fit to an *a priori* constraint on the solution:

$$\hat{x}\left(k\right)=\underset{x\left(k\right)}{arg\;min}\;\left\{{\left\|Ax\left(k\right)-y\left(k\right)\right\|}_{2}^{2}+\lambda_{k}^{2}{\left\|Lx\left(k\right)\right\|}_{2}^{2}\right\}\;,$$

where *λ*_*k*_ is a regularization parameter that controls the trade-off between the residual norm $\left({\left\|Ax\left(k\right)-y\left(k\right)\right\|}_{2}\right)$ and the constraint norm $\left({\left\|Lx\left(k\right)\right\|}_{2}\right)$ , and **L** is a regularization matrix based on the chosen constraint. In this study, **L** is chosen to be the identity matrix (zero-order Tikhonov). At each time, the *λ*_*k*_ value was first determined by using the L-curve method (Hansen, 2001). The final solution was computed by using a single *λ* value equal to the median of all *λ*_*k*_ values over time.

The origins of PVCs were estimated from the activation times (ATs). These ATs were computed using a spatiotemporal (ST) method proposed in Erem et al. (2014a). In this approach, first, the local activation times (LATs) from inversely reconstructed electrograms (EGMs) were estimated at each node as the time instant of its minimal derivative. However, these LATs are affected by noise in the EGM reconstructions (Erem et al., 2014a; Cluitmans et al., 2017; Rasoolzadeh et al., 2022). To avoid spatial inconsistencies in the neighboring LATs, the ST method regularizes the derivative-based AT values so that they are smoothed over space. Finally, the PVC origin was marked as the node with the earliest AT during the QRS interval.

### 2.4 Summary of ECGI pipelines

**FIGURE 2:**
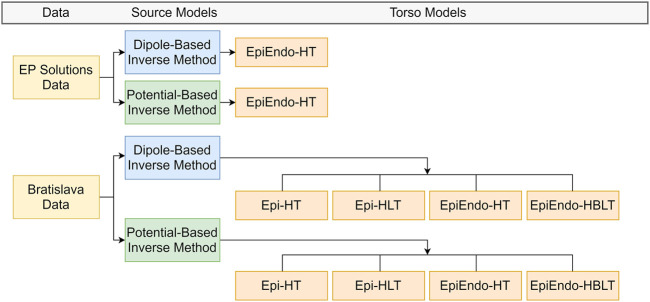
Summary of the ECGI pipelines used in this study. EP-Solutions data set geometry is only available in terms of the EpiEndo heart surface with the homogeneous torso. The Bratislava data set has both the Epi and EpiEndo surfaces defined, as well as inhomogeneities (lungs and intracavitary blood), as described in Section 2.4.

All ECGI cases evaluated in this study are summarized in Figure 2. A detailed description of each pipeline is given as follows.

For the EP-Solutions data, only the heart (EpiEndo) and torso meshes were available. Therefore, for this data set, only the homogeneous heart–torso (HT) model was considered with the EpiEndo surface (*i.e.*, EpiEndo–HT).

For the Bratislava data, the models of the lungs and blood were also available. Therefore, homogeneous heart–torso (HT) and inhomogeneous heart–torso models were used for computation. The homogeneous heart–torso model was computed for both Epi (Epi–HT) and EpiEndo (EpiEndo–HT) surfaces. The inhomogeneous heart–torso model has two different scenarios. For the Epi surface mesh, the lungs are outside the closed heart surface; thus, the Epi heart surface solutions were obtained for a forward model including only the lungs as the torso inhomogeneity (Epi-HLT model). For the EpiEndo surface mesh, the intracavitary blood is also outside the closed heart surface; thus, we included blood as well as the lungs as the torso inhomogeneities for the EpiEndo surface solutions (EpiEndo–HBLT). The conductivity values of the lungs and blood were assumed to be four times lower than the conductivity values of the torso and three times higher than the conductivity values of the torso, respectively. The homogeneous and inhomogeneous forward transfer matrices **T** and **A** between the heart and torso surface meshes from Eqs 1, 4, respectively, were first calculated for the fine torso mesh. Then, a reduced transfer matrix that relates the heart potentials only to the measured points on the torso was obtained by bilinear interpolation of the values from the vertices of the triangle corresponding to the position of the measuring electrode.

Finally, the inverse problem was solved and the PVC origin was estimated for both the source models, as introduced in Section 2.3.1 and Section 2.3.2 for each case. This ECGI pipeline results in four different solutions for each source model (dipole or heart surface potential): Epi-HT and Epi-HLT for sources on the Epi surface and EpiEndo-HT and EpiEndo-HBLT for sources on the EpiEndo surface (see Figure 2).

### 2.5 Evaluation methods

Quantitative evaluations are carried out using the PVC localization error (LE) metric, defined as the Euclidean distance between the ground truth and the estimated pacing site or PVC origin.

The EP Solutions data set includes single-paced (from LV or RV) data from five patients with previously implanted CRT devices and biventricular pacemaker leads. The positions of these pacing leads obtained from CT scans are provided as the ground truth pacing site in the data set.

For the Bratislava data set, one to three nodes were manually marked on the EpiEndo heart geometry of each patient as the true PVC origins (the position of the successful ablation site) by the physician who performed the RFA procedure, according to the RFA procedure report. If there is more than one PVC origin for a patient, the center of gravity of all annotated locations for that patient is defined as the single (mean) ground truth PVC origin for that patient, and the LE is calculated with respect to this single (mean) annotation.

RRE and AT maps over the heart surfaces were also qualitatively evaluated to assess the PVC localization performance of each source model and each torso model.

## 3 Results

**FIGURE 3:**
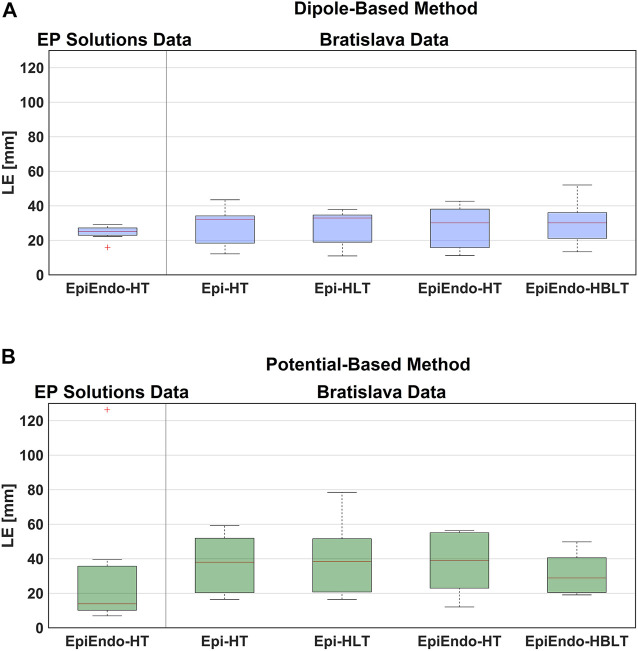
Boxplot representations of the LE values obtained for both data sets, both source models [dipole-based **(A)** and potential-based **(B)**], and all heart–torso models, for all patients. The red line in the boxplots corresponds to the median LE value for all patients, and the upper and lower boundaries of the boxes correspond to the third (Q3) and the first (Q1) quartiles, with IQR = Q3-Q1.

The overall characteristics of accuracy for both data sets and cardiac source models (dipole/potential) and all heart–torso models (Epi/EpiEndo heart surface and homogeneous/inhomogeneous torso) are provided in Table 2. This table lists the LE range (minimum–maximum), mean ± standard deviation (mean ± std), and median with the interquartile range (med (IQR)) values of all patients for each case under investigation. In addition, the ratio of the correctly identified ventricle (LV or RV) of the pacing site/PVC origin of all patients is given for each case (denoted as “TCR” in the table). Boxplots for visualizing the summary statistics for all cases evaluated in this study are also presented in Figure 3. The information presented in Table 2 and Figure 3 is based on individual LE values and classifications of the pacing site/PVC origin given in Table 3 for the EP-Solutions data set and in Tables 4–7 for the Bratislava data set. It should be noted that for P5, the ablation was applied on both sides of the septum (indicated as “septum (LV/RV)” in Table 1). Since the methods employed for PVC localization in this study select only a single point as the PVC origin estimate, both LV and RV origins were counted as correct ventricle estimation.

**TABLE 2:** Overall characteristics of accuracy for both data sets and cardiac source models and all heart–torso models. All LE values are in millimeter. ‘TCR’ stands for the ‘true classification ratio’, which is defined as the number of correctly identified ventricles (LV or RV) as the pacing site/PVC origin over the number of all available patients.

Data set	Characteristics of accuracy	Dipole-based	Potential-based	Dipole-based	Potential-based
		Epi-HT		EpiEndo-HT	
	Range (min–max)	NA	NA	16.0–29.2	7.0–126.3
EP-Solutions data set	Mean ± std	NA	NA	24.5 ± 4.1	31.6 ± 40.0
	Med (IQR)	NA	NA	25.2 (3.3)	13.9 (22.9)
	TCR	NA	NA	6/8	7/8
		Epi–HT		EpiEndo–HT	
	Range (min–max)	12.2–43.5	16.5–59.2	11.2–42.7	12.-56.5
	Mean ± std	27.5 ± 10.5	37.2 ± 15.9	27.9 ± 11.7	37.6 ± 17.8
	Med (IQR)	32.3 (15.5)	38.1 (27.2)	30.2 (20.0)	39.2 (31.7)
	TCR	9/10	5/10	9/10	9/10
Bratislava data set		Epi–HLT		EpiEndo–HBLT	
	Range (min–max)	11.0–37.9	16.5–78.5	13.4–52.1	19.1–49.8
	Mean ± std	27.4 ± 9.7	38.6 ± 18.9	29.5 ± 11.1	31.3 ± 11.1
	Med (IQR)	33.0 (14.7)	38.6 (26.7)	30.2 (13.9)	28.9 (19.1)
	TCR	9/10	8/10	9/10	9/10

**TABLE 3:** Localization errors (mm) of the EP-Solutions data set for both source models. The incorrectly estimated ventricle is indicated with italic and bold font.

Patient #	Pacing side	Dipole-based	Potential-based	Dipole-based estimated side	Potential-based estimated side
P24	LV	16.0	11.6	LV	LV
P24	RV	29.2	31.9	RV	RV
P26	LV	25.6	8.9	LV	LV
P26	RV	24.8	39.6	***LV***	RV
P27	RV	23.7	12.2	RV	RV
P33	LV	28.4	126.3	LV	***RV***
P33	RV	22.3	15.6	RV	RV
P36	RV	26.1	7.0	***LV***	RV

**TABLE 4:** Localization errors (mm) of the Bratislava data set for the homogeneous heart–torso model on the Epi surface (Epi-HT). The incorrectly estimated ventricle is indicated with italic and bold font.

Patient #	Ablation side	Dipole-based	Potential-based	Dipole-based estimated side	Potential-based estimated side
P1	RVOT	12.2	27.9	RV	RV
P2	RVOT	35.5	58.2	RV	***LV***
P3	RVOT	18.7	20.4	RV	***LV***
P4	RVOT	32.8	20.0	RV	RV
P5	Septum (LV/RV)	34.2	59.2	LV	LV
P6	RV	43.5	16.5	***LV***	***LV***
P7	LV	18.4	42.1	LV	LV
P8	RVOT	14.8	36.2	RV	***LV***
P9	RVOT	33.3	51.9	RV	***LV***
P10	LV	31.7	39.9	LV	LV

**TABLE 5:** Localization errors (mm) of the Bratislava data set for the homogeneous heart–torso model on the EpiEndo surface (EpiEndo–HT). The incorrectly estimated ventricle is indicated with italic and bold font.

Patient #	Ablation side	Dipole-based	Potential-based	Dipole-based estimated side	Potential-based estimated side
P1	RVOT	11.9	29.2	RV	RV
P2	RVOT	36.6	55.2	RV	RV
P3	RVOT	11.2	23.0	RV	***LV***
P4	RVOT	30.8	12.1	RV	RV
P5	Septum (LV/RV)	38.9	49.15	LV	RV
P6	RV	42.7	23.3	***LV***	RV
P7	LV	23.7	53.7	LV	LV
P8	RVOT	15.8	56.5	RV	RV
P9	RVOT	29.5	19.1	RV	RV
P10	LV	38.1	55.1	LV	LV

**TABLE 6:** Localization errors (mm) of the Bratislava data set for the inhomogeneous heart–torso model (with lungs) on the Epi surface (Epi–HLT). The incorrectly estimated ventricle is indicated with italic and bold font.

Patient #	Ablation side	Dipole-based	Potential-based	Dipole-based estimated side	Potential-based estimated side
P1	RVOT	11.0	27.7	RV	RV
P2	RVOT	35.1	51.6	RV	RV
P3	RVOT	19.0	20.8	RV	RV
P4	RVOT	32.8	20.0	RV	RV
P5	Septum (LV/RV)	33.1	40.7	LV	RV
P6	RV	37.9	16.5	***LV***	***LV***
P7	LV	14.9	42.0	LV	LV
P8	RVOT	21.7	78.5	RV	RV
P9	RVOT	33.3	51.9	RV	***LV***
P10	LV	34.7	36.4	LV	LV

**TABLE 7:** Localization errors (mm) of the Bratislava data set for the inhomogeneous heart–torso model (with lungs and blood) on the EpiEndo surface (EpiEndo–HBLT). The incorrectly estimated ventricle is indicated with italic and bold font.

Patient #	Ablation side	Dipole-based	Potential-based	Dipole-based estimated side	Potential-based estimated side
P1	RVOT	13.4	29.2	RV	RV
P2	RVOT	36.6	40.6	RV	RV
P3	RVOT	19.9	19.1	RV	RV
P4	RVOT	32.3	28.6	RV	RV
P5	Septum (LV/RV)	33.0	39.6	LV	RV
P6	RV	36.1	23.4	***LV***	RV
P7	LV	21.2	49.8	LV	LV
P8	RVOT	22.1	20.5	RV	RV
P9	RVOT	28.0	19.2	RV	RV
P10	LV	52.1	42.9	LV	***RV***

### 3.1 EP-Solutions data set

The EP-Solutions data from the EDGAR database only include the homogeneous heart–torso geometric model with the heart mesh represented in terms of the EpiEndo surface.

Based on the summary statistics in Table 2, the dipole-based solutions have a mean and median LE of 24.5 and 25.2 mm, respectively, whereas, for the potential-based solutions, the mean and median LE values are 31.6 and 13.9 mm, respectively. On close inspection of detailed results for the potential-based solution in Table 3, one can observe that five out of eight LE values are less than 16 mm. The reason for the high mean value and large variability across patients indicated by the high standard deviation and IQR values is due to one outlier case (P33-LV) with an LE value of 126.3 mm. When we exclude the outlier from the summary statistics of the potential-based solution, its mean LE decreases to 18.1 mm, whereas the median LE value does not change dramatically (becomes 12.2 mm). However, the variability across patients decreases significantly (std of 12.5 mm; IQR of 13.5 mm, with a range of 7.0–39.6 mm).

The boxplots in Figure 3 for the dipole-based and potential-based solutions show a higher IQR value for the potential-based solutions, including the outlier. Despite a higher median LE value with the dipole-based solutions than that of the potential-based solutions, the former is more stable, with an IQR value of 3.3 mm, which is much smaller than the IQR value of the potential-based counterparts, even excluding the outlier.

The dipole-based solution finds 6/8 pacing sites in the correct ventricle, where P26-RV and P36-RV are the incorrectly classified cases. This ratio is 7/8 for the potential-based solution, where the incorrect classification corresponds to the outlier case (P33-LV).

#### 3.1.1 Qualitative evaluation of the EP-Solutions data set results

**FIGURE 4:**
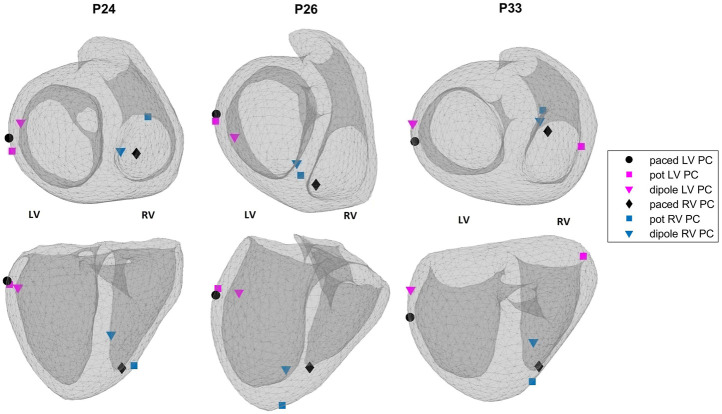
Ground truth pacing locations (black marks: circle—LV; diamond—RV) and the estimated pacing locations for the potential-based (squares) and the dipole-based (triangles) source models on the EpiEndo geometries for the EP-Solutions data set. Different colors are used to denote LV and RV pacing site estimates (blue—RV and magenta—LV). Top row: base to apex view and bottom row: posterior view of the heart. The left and right ventricles for the geometries in both rows are labeled as LV and RV, respectively.

Figure 4 shows the EpiEndo heart geometries from two views, ground truth pacing locations for the LV- and RV-paced beats and the corresponding localization estimates by both source models for three patients for which there are both LV- and RV-paced beats (total of 6/8 cases). In P24, both source models correctly classify the correct pacing ventricle, with varying LE values. P26 and P33 are chosen to represent patients for which estimations by the dipole-based and potential-based solutions find the pacing site in the wrong ventricle in one of the two pacing sites for each patient (see Table 3). On close inspection of P26-RV, it is visible that the incorrect result with the dipole-based solution is close to the true pacing site, but on the opposite side of the septum. For the same case, the potential-based solution estimates the pacing origin in the correct ventricle (RV), but on the epicardium instead of the endocardium. The incorrectly classified outlier pacing site estimate for P33-LV with the potential-based solution ended up in the wrong ventricle, at a significant distance from the true pacing site.

### 3.2 Bratislava data set

For this data set, we have considered homogeneous and inhomogeneous torso models, and each torso model was used in conjunction with the Epi surface and EpiEndo surface representation. Thus, the results are presented separately for each case as follows.

#### 3.2.1 Homogeneous heart–torso models

##### 3.2.1.1 Epi surface

The summary statistics in Table 2 show that the dipole-based solutions have a mean and median LE of 27.5 and 32.0 mm, respectively, whereas the mean and median LE values for the potential-based solutions are 37.2 and 38.1 mm, respectively, and larger than those for the dipole-based model. The potential-based solutions are also more variable across patients, with an IQR value almost two times higher than that of the dipole-based solutions (see Figure 3). With this heart–torso model, out of 10 patients, the PVC origins of nine patients were localized in the correct ventricle with the dipole-based model. However, with the potential-based model, this number decreased to 5.

##### 3.2.1.2 EpiEndo surface

The mean and median values given in Table 2 are 27.9 and 30.2 mm, respectively, for the dipole-based model and 37.6 and 39.2 mm, respectively, for the potential-based model. In terms of the mean and median LE values, the performances of both source models on the EpiEndo surface are similar to those of their counterparts on the Epi surface. However, the variability of the results with both source models is higher in the EpiEndo surface than in the Epi surface, as indicated by the higher std and IQR values in the former (also see Figure 3). Still, both source models estimated the PVC origins in the correct ventricle for nine patients out of 10 with this heart–torso model.

#### 3.2.2 The effects of torso inhomogeneities

##### 3.2.2.1 Epi surface

As observed in Table 2, the dipole-based solutions have a mean and median LE of 27.4 and 33.0 mm, respectively. The mean and median LE values for the potential-based solutions are both 38.6 mm. The variability of the potential-based LE values is higher than that of the dipole-based LEs, as is evident from the std and IQR values in the tables and the boxplots in Figure 3. It should be noted that there is no distinguishable difference between the Epi-HT and Epi-HLT results with both source models, except a higher range of LE values (larger maximum LE value) obtained with the potential-based model in the Epi-HLT case. With this heart–torso model, the dipole-based source model still estimated the correct ventricle for the PVC origins for nine out of 10 patients; it was 8/10 for the potential-based source model.

##### 3.2.2.2 EpiEndo surface

The mean and median values are 29.5 and 30.2 mm, respectively, for the dipole-based model and 31.3 and 28.8 mm, respectively, for the potential-based model (Table 2). In terms of the mean and median LE values, the performance of the dipole-based model is similar to that of the inhomogeneous case, Epi-HLT, and the homogeneous case, EpiEndo-HT. However, adding the inhomogeneities and solving the problem on the EpiEndo surface improves the potential-based solutions, compared to all remaining heart–torso models. The mean LE decreases by 19%–18% compared to the inhomogeneous Epi-HLT and homogeneous EpiEndo-HT, respectively. There is a more dramatic improvement in the median LE values, indicated by a 25%–26% decrease compared to the inhomogeneous Epi-HLT and homogeneous EpiEndo-HT, respectively. With the dipole-based solutions, EpiEndo-HBLT has a comparable IQR value to both Epi surface results and a smaller IQR value than that of the homogeneous EpiEndo-HT solution (tighter boxplot in Figure 3 with EpiEndo–HBLT than in EpiEndo–HT). The variability of the potential-based EpiEndo–HBLT solutions is much less than that of the other heart–torso models, as indicated by a smaller IQR value and tighter boxplot in Figure 3. Similar to the homogeneous EpiEndo–HT solution, both source models estimated the PVC origins in the correct ventricle for nine out of 10 patients.

#### 3.2.3 Qualitative evaluation of the Bratislava data set results

**FIGURE 5:**
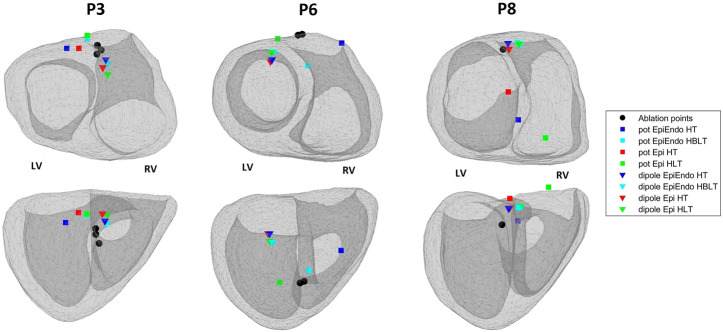
Ablation points (black circles) and the estimated pacing locations for the potential-based (squares) and the dipole-based (triangles) source models on the EpiEndo geometries for the Bratislava data set. Different colors are used to denote different heart–torso geometries used in this study. Top row: base to apex view and bottom row: posterior view of the heart. The left and right ventricles for the geometries in both rows are labeled as LV and RV, respectively.

Out of ten Bratislava patients, three cases are selected to display in Figure 5. This figure shows the EpiEndo heart geometries from two views, ground truth PVC origins as marked by the physician and the corresponding localization estimates of all torso models and source models. P3 localization estimates are good in terms of mean LE for all heart–torso models for both source representations (17.2 and 20.8 mm for the dipole and potential-based models, respectively). P6 illustrates a case where the potential-based solution outperforms the dipole-based solution (mean LE over all heart–torso models: 40.1 and 19.9 mm for the dipole and potential-based models, respectively), and P8 illustrates the opposite case (mean LE over all heart–torso models: 18.6 and 51.9 mm for the dipole and potential-based models, respectively).

For P3, despite small LE values with both source models, the PVC origin is estimated in the wrong ventricle with the homogeneous heart–torso models (Epi-HT and EpiEndoHT) with the potential-based source model. For P6, although the mean LE value over the four torso models is 40.1 mm for the dipole-based solution, all estimates are clustered in the same area, with a standard deviation value of 3.1 mm. However, they are all found in the wrong ventricle. For the potential-based model, despite a small LE value of 16.5 mm with the Epi-HT, the PVC origin estimate is in the wrong ventricle. For P8, the mean LE value for the potential-based solution over the four torso models is 51.9 mm, with the worst result (78.5 mm) for the Epi-HLT case, but the PVC origin estimate is in the correct ventricle. However, for the Epi-HT model, the PVC origin estimate is in the wrong ventricle, despite a smaller LE value. There is high variability in the localization results with the potential-based model for this patient, which is also apparent from a high LE standard deviation value of 21.8 mm over all torso models.

**FIGURE 6:**
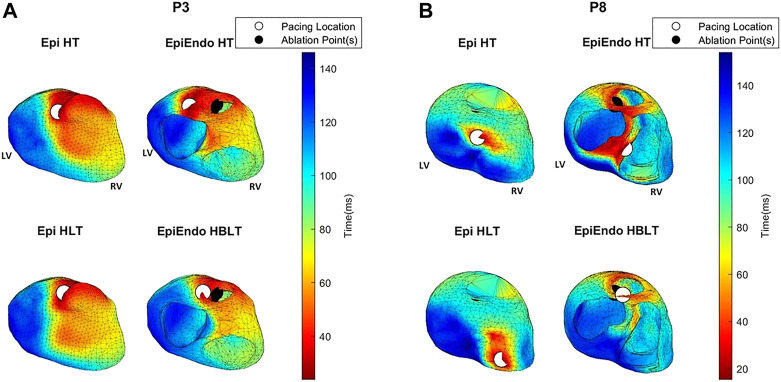
AT maps corresponding to the potential-based solutions for P3 **(A)** and P8 **(B)** of the Bratislava data set, all with the same view angle, including the estimated pacing location (white circles) and the ground truth PVC origin (black circles). Note that the ground truth PVC origins are marked on the EpiEndo surface; thus, the corresponding marking is not always visible on the Epi surface if the ablation point is on the endocardium.

**FIGURE 7:**
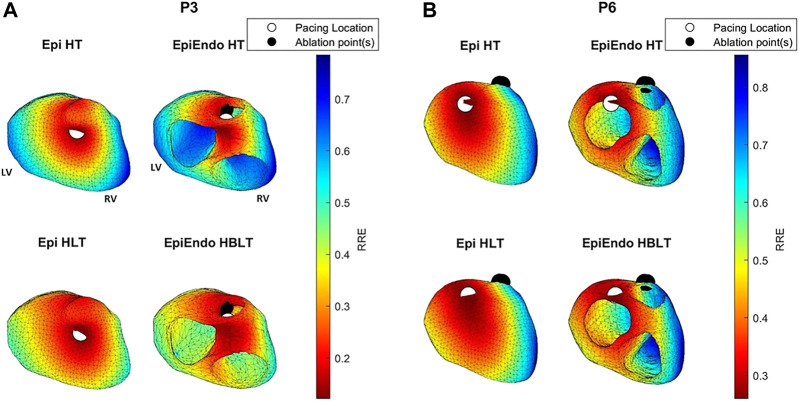
RRE maps corresponding to the dipole-based solutions for P3 **(A)** and P6 **(B)** of the Bratislava data set, all with the same view angle, including the estimated pacing location (white circles) and the ground truth PVC origin (black circles). The PVC origin markings are similar to those in Figure 6.

The distribution of the criteria function values that we use for localization of PVCs for both inverse methods (AT for the potential-based and RRE for the dipole-based) is presented in Figures 6, 7. For both source models, examples of one good and one bad performing patient are selected from the localization results in Figure 5. From these figures, it is also apparent that the inverse solution is not unique, and similar values of the criteria functions are observed in a larger area of the heart model.

For P3, both the AT maps and the RRE maps for all models are consistent with each other with similar distributions, and with the ground truth, hence explaining the lower LE values for this patient. The AT maps for P8 are quite different in the Epi versus EpiEndo solutions. The AT maps for the Epi solutions are not consistent with the ground truth PVC location. For the EpiEndo solutions, the AT maps computed in the homogeneous and inhomogeneous torso are not the same. Only the EpiEndo–HBLT model, having the smallest LE value among the four torso models (20.5 mm), reflects the expected AT distribution for the ground truth. The EpiEndo–HT model resembles the homogeneous case near the ground truth PVC location but has another large region of smaller AT values throughout the septum, incorrectly estimating the PVC location. The RRE maps for P6 are quite consistent for all four methods, with similar RRE distributions, but lower RRE values are not observed in a region consistent with the location of the ground truth PVC origin.

## 4 Discussion

In this paper, the pacing site/PVC origin localization performances of two widely used source models in ECGI (dipole-based and potential-based) were compared using the same clinical data. The BSPs were measured on patients via 240 or 128 electrodes placed on the whole torso. For each patient, the specific torso geometry with the electrode locations and the heart position, and the inhomogeneities, if applicable, were provided from the CT scan.

The main contributions of this study can be summarized as follows:

• Pacing site/PVC origin localization has been a major clinical aim of noninvasive ECGI. Different cardiac equivalent source models have been used with variable localization accuracy. This study demonstrated that the dipole-based model gives a more robust localization performance than the potential-based model with the zero-order Tikhonov regularization solutions.
• These source models were evaluated with data from CRT patients paced by an implanted electrode and data consisting of spontaneously occurring PVCs in patients who underwent successful RF ablation procedures. Several studies have evaluated PVC localization accuracy with paced data, but only a limited number of ECGI studies focus on finding spontaneous PVC origins with clinical data.
• Several studies explored the effects of torso inhomogeneities on ECGI reconstructions with conflicting results, and studies focusing on spontaneous PVC origin localization performance are lacking, demonstrating the need for further investigation. This study showed that the torso inhomogeneities affect the performances of PVC origin localization in each source model differently. While the dipole-based solutions were, in general, robust to different heart–torso models, the potential-based solutions produced the best results on the EpiEndo surface with the heart–torso model, including the lungs and the blood. Hence, care must be taken in generating patient-specific geometric and forward models depending on the source model representation used in ECGI.

### 4.1 Comparison of dipole-based and potential-based solutions

The dipole-based source model was robust across data sets and different heart–torso models, with mean and median LE values differing by 5.0–7.8 mm. This range was even smaller (differing by 2.0–2.8 mm) for the Bratislava data set. The mean and median LE values for the potential-based source model, on the other hand, varied with higher margins of 7.3–10.3 mm differences even across various heart–torso models of the Bratislava model. The median LE of the potential-based model for the EP-Solutions data set was smaller than that of the Bratislava data set results. In general, for the EP-Solutions data set, the LE values (excluding the outlier) with the potential-based source model were smaller than those of the dipole-based model. On the other hand, the dipole-based LE values had smaller variability.

The main reason for the conflicting results with the potential-based solutions could be the many steps employed in this approach, requiring first reconstructing the heart surface potentials, then estimating and smoothing the ATs, and finding the node with the smallest AT value for PVC localization. Despite its widespread application for solving inverse problems, including the ECGI, the zero-order Tikhonov regularization spatially smooths the reconstructed potentials. As a result, the wavefront on the isopotential map at a single time instant appears spread over a larger area, with the effect of several points appearing to be activated around the same time. The spatiotemporal AT estimation method used in this study also introduces errors. Schuler et al. evaluated this algorithm and several others based on extracellular potential- (Epi and EpiEndo) and transmembrane potential-based solutions (Schuler et al., 2022). Their results confirmed that the spatiotemporal method is superior to using only the time derivative of EGMs, but were prone to line-of-block artifacts when applied to the Epi and EpiEndo potential reconstructions. They reported that these artifacts are mainly caused by the inherent spatial smoothing effect of the inverse reconstruction. Thus, the spatial smoothing introduced by the Tikhonov regularization methods, coupled with errors introduced with the AT estimation, could result in incorrectly estimating the pacing site/PVC origin location in our study.

There is a wide variation in the localization performances of different ECGI methods reported in the literature, with median/mean LE values ranging from 5.0 to 43.0 mm. Our median LE values for both data sets ranging from 25.2 to 33.0 mm (30.2–33.0 mm with the Bratislava data set) and 13.9–39.2 mm (28.9–39.2 mm with the Bratislava data set) using the dipole-based and potential-based source models, respectively, fall within this reported range. Some specific relevant studies from the literature are summarized here to give a perspective to our results. On a clinical data set of paced beats, Erem et al. found median LE values of 30.5 mm (non-septal) and 43.0 mm (septal) for the potential-based solutions, using a spline-based inverse method and the same AT estimation used in this study (Erem et al., 2014a). Zhou et al., using the same clinical data set, improved these median LE values to 13.4 mm (non-septal) and 27.6 mm (septal) with the EDL source model, using the sparse Bayesian learning method (Zhou et al., 2019). As an alternative to solving the conventional inverse ECG problem, Sapp et al. used a regression method, which is trained with known pacing locations and the corresponding BSPs (Sapp et al., 2017). They reported a mean (geodesic distance-based) LE of 12 ± 8 mm. Later, using a similar approach, Zhou et al. found a mean LE of 6.5 ± 2.6 mm for 25 induced VTs and 5.9 ± 2.6 mm for 26 VT exit and PVC origin sites (Zhou et al., 2021). Potyagaylo et al. used the fastest route algorithm (FRA) and its hybrid derivations. They found median LE values between 9 and 28 mm, where the largest value corresponded to single dipole fit, without using the FRA, and the smallest value combined single dipole fit with FRA (Potyagaylo et al., 2019a). In another study, they combined FRA with dynamic time warping and reduced the median LE values from 16 mm in the FRA method to 5 mm (Potyagaylo et al., 2019b). This last study is from the same experiment for the EDGAR EP-Solutions data set; however, they have included the torso inhomogeneities of the lungs and ventricular blood masses and included 10 patients, while the EDGAR data set includes only a subset of their data and only the homogeneous torso model.

With respect to the potential-based results in Erem et al. (2014a), which proposed the AT algorithm that we used in this study, the Bratislava data set results were comparable, and the EP-Solutions data set results were better. Our dipole-based solutions were comparable to the results in Potyagaylo et al. (2019a) with the single dipole fit, especially with the EP-Solutions data set. Our LE values were larger with the Bratislava data set. Our LE values were worse than those of the direct PVC reconstruction methods such as linear regression in Zhou et al. (2021) or the simulation-based FRA method that iteratively reconstructs the ATs from the BSPs (Potyagaylo et al., 2019b; Potyagaylo et al., 2019a).

### 4.2 Effects of various heart–torso models

In this section, we discuss the results corresponding to the four different heart–torso models of the Bratislava data set.

As we stated previously, the mean/median LE values for the dipole-based source model do not vary significantly across different heart–torso models. However, the lowest median LE value (30.2 mm) with the smallest variation (IQR of 11.1 mm) was obtained for the EpiEndo–HBLT case (also see Figure 3). On the other hand, the other heart–torso models closely followed in performance. Thus, a homogeneous model could be used with this source model due to its simplicity compared to an inhomogeneous model. PVCs originating in the septum can be better identified on the EpiEndo surface; therefore, EpiEndo–HT could be a good choice for localizing the PVC origins by a single dipole.

The LE values for the potential-based solution were usually larger and more varying across different heart–torso models than the dipole-based results. The best results for this source model were obtained with the EpiEndo–HBLT model, with a median LE of 28.9 mm and an IQR of 19.1 mm, which is also observed in the boxplots of Figure 3. These results suggest that the EpiEndo–HBLT model can be preferred in PVC localization applications if an accurate inhomogeneous model is available. Otherwise, EpiEndo–HT could be preferred since there is not much difference between the solutions on the Epi and EpiEndo surfaces in the homogeneous torso models, but the PVC can occur in the endocardium as well as in the epicardium.

When we compared our work with similar studies in the literature, including the lungs in the Epi surface representation did not have a significantly noticeable effect on our results, whereas other studies found the lungs as a significant inhomogeneity (van Oosterom, 2014). Including blood for the EpiEndo surface representation had a noticeable effect on our results, even more so for the potential-based source model. This result is consistent with those of studies indicating blood as an important inhomogeneity in the forward models. However, while including blood improved the results in Keller et al. (2010), it had the opposite effect in Potyagaylo et al. (2016), where including blood in the model increased the appearance of outliers in the LE. We observed a similar increase in the outliers with the dipole-based model, but not with the potential-based model.

### 4.3 Pacing site versus spontaneous PVC origin localization

To have a fair comparison between the two data sets, we only consider the homogeneous torso model with the EpiEndo heart surface in this discussion since that is the only available geometry in the EP-Solutions data set. We obtained smaller mean and median LE values with the EP-Solutions data set than with the Bratislava data set. This difference between data sets was even more pronounced with the potential-based solutions.

While the position of the stimulating electrode for the EP-Solutions data set was known exactly from the CT scan, for the Bratislava data set, the position of the successful RFA intervention was annotated by the physician as a vertex (or vertices) of the triangles of the EpiEndo heart surface (post facto) according to the documentations from the procedure. Therefore, the accuracy of such ground truth PVC location estimation can be influenced by a model error since the mean distance between the vertices on the EpiEndo surfaces was 4.7 ± 0.6 mm for the Bratislava data set. In addition, there is a model error between the ground truth PVC origin (*i.e.*, the center of the annotated ablation points) on the EpiEndo surface and the vertices (possible PVC origins) on the Epi and EpiEndo surfaces. This error is quantified in Table 8, which lists the smallest distance between the ground truth PVC origin and the vertices of the Epi or EpiEndo surface mesh for all patients in the Bratislava data set. If only one ablation point was assigned (as in P1 and P2), then this error for the EpiEndo surface is 0. It is larger for the Epi surface, especially for patients with ablation points near or on the septum. Finally, some errors can be due to the subjective selection of the ground truth PVC origins by the evaluator. The bilinear interpolation that is used to calculate the forward matrix rows corresponding to the electrode coordinates could be another model error. Molero et al. showed that when the electrode positions matched the torso nodes, ECGI performance during atrial fibrillation was better than that when taking the nearest torso node as the electrode position (Molero et al., 2022). The effects of torso mesh construction and electrode positions with respect to the torso nodes should be further evaluated in future studies for PVC origin localization with the Bratislava data set. These model errors are geometry-related and do not depend on a specific source model.

**TABLE 8:** Smallest distance (mm) between the center of the annotated ablation points (*i.e.*, the ground truth PVC origin) and the vertices of the Epi and EpiEndo heart surface meshes for all patients in the Bratislava data set.

	P1	P2	P3	P4	P5	P6	P7	P8	P9	P10
Epi	7.0	6.7	12.1	10.2	4.2	2.6	4.8	6.8	2.5	3.8
EpiEndo	0.0	0.0	2.6	1.5	3.9	1.7	2.5	0.0	0.6	0.5

There are also some differences between the mechanisms of paced activation and spontaneous PVC. As was mentioned previously in the case of the paced activation with the EP-Solutions data set, the position of the pacing electrode was known exactly from the CT scan, and the BSP measurement was performed simultaneously with the pacing. In the Bratislava data set with the spontaneous PVCs, the BSP measurements were performed earlier, separately from the RFA procedure. Although we did not observe any correlation between LE values and the time-to-intervention, the mechanisms for PVCs are more complex than those of pacing via implanted electrodes. A PVC focus can have multiple exits or preferential exit sites (to the right or left ventricle) at a particular time. During the catheter ablation, the physicians can eliminate one exit site in a certain location, but PVC could remain still present with a slightly different morphology. The procedure is continued, focusing on finding “the true focus,” if possible, or the site with the smallest distance from the focus or the site that is best accessible (not necessarily the closest location to the true source). Therefore, the definition of the ground truth and, thus also the LE in the case of the spontaneous PVC, can be less precise than for the paced stimulation.

### 4.4 Limitations of the study

In this study, only the Tikhonov zero-order regularization method was considered for the potential-based source model, which has its limitations, as discussed in Section 4. Other more robust and/or edge-preserving ECGI methods such as statistical estimation techniques (Serinagaoglu et al., 2006; Aydin and Dogrusoz, 2011; Erenler and Serinagaoglu Dogrusoz, 2019), *L*_0_-norm-, *L*_1_-norm-, and *L*_*p*_-norm (1 ≤ *p* ≤ 2)-based methods (Ghosh and Rudy, 2009; Xu et al., 2014; Wang et al., 2016; Rahimi et al., 2013; Rahimi et al., 2016), and parametric or nonparametric spline fitting (Erem et al., 2014a; Onak et al., 2019; Onak et al., 2022) could reconstruct sharper wavefronts. Alternatively, potential-based source models (e.g., reconstructions in terms of transmembrane potentials (Schuler et al., 2022)) or other more novel PVC localization methods that have been shown to have more accurate PVC localization with smaller LE values (Sapp et al., 2017; Potyagaylo et al., 2019b; Potyagaylo et al., 2019a; Zhou et al., 2021) could be evaluated.

Here, similar to many studies in the literature, we used the Euclidean distance to define the LE (Erem et al., 2014a; Zhou et al., 2019; Zhou et al., 2021; Potyagaylo et al., 2019b; Potyagaylo et al., 2019a). Despite its popularity, this definition of the LE could be misleading in some cases, most significantly when the PVC originates in the septum. The Euclidean distance-based LE could still be small, but the PVC estimate could be in the incorrect ventricle, which would mislead the physician about the ventricle at which the RFA procedure should be started. Alternatively, the geodesic distance-based LE definition would be more appropriate and would produce larger LEs for the aforementioned case (Sapp et al., 2017; Pilia et al., 2022). Comparing the performances of the two LE definitions was not within the scope of this study, but this limitation should be considered in future studies.

Although we evaluated the effects of homogeneous and inhomogeneous forward models on pacing site/PVC origin localization in this study, this was by no means an extensive evaluation. Our models included only the lungs with the Epi surface representation and both the lungs and blood with the EpiEndo surface. Other studies in the literature looked into the effects of more complicated heart–torso geometries, including some combination of the anisotropic skeletal muscle, subcutaneous fat, bones, lungs, and intracavitary blood, on the forward and inverse ECG solutions (Klepfer et al., 1997; Ramanathan and Rudy, 2001a; Keller et al., 2010; Bear et al., 2015; Punshchykova et al., 2016; Zemzemi et al., 2016). The general conclusion is that the BSP maps were found to be similar using various torso models, but the detailed inhomogeneous models produced potential amplitudes closer to the true potentials, with blood and the anisotropic skeletal muscle being the most influential tissues. More recently, the liver tissue has also been included as a significant inhomogeneity in addition to the other tissues (Potyagaylo et al., 2016; Doste et al., 2020; Schuler et al., 2022). Thus, future work could focus on the systematic evaluation of the effects of individual tissues on PVC localization with various source models.

## 5 Conclusion

The dipole-based source model is more robust and can be preferred over the potential-based source model for pacing site/PVC origin localization. However, the dipole-based solutions cannot provide a detailed progression of the electrograms/potential maps over the entire QRS region or the T-wave (repolarization-related arrhythmias). Then, the potential-based solutions would be suitable. However, in that case, the method of inverse solution and AT estimation should be chosen carefully to preserve sharp changes on the wavefronts of the isopotential maps and to avoid artifacts (extensive spatial smoothing or line-of-block artifacts) in the AT maps. Direct solutions in terms of PVC locations (Sapp et al., 2017; Doste et al., 2020; Zhou et al., 2021; Pilia et al., 2022) or ATs (Erem et al., 2014b; Potyagaylo et al., 2019b; Potyagaylo et al., 2019a) could also improve the localization results.

Homogeneous torso models as well as the inhomogeneous torso models could be used with the dipole-based solutions. However, inhomogeneous torso models could be preferred for the potential-based solutions if an accurate torso model is available.

## Data Availability

The raw data supporting the conclusions of this article will be made available by the authors, without undue reservation.
